# Negative Effects of Occurrence of Mycotoxins in Animal Feed and Biological Methods of Their Detoxification: A Review

**DOI:** 10.3390/molecules29194563

**Published:** 2024-09-25

**Authors:** Michał Lach, Katarzyna Kotarska

**Affiliations:** Department of Distillery Technology and Renewable Energy, Prof. Wacław Dąbrowski Institute of Agriculture and Food Biotechnology—State Research Institute, Powstańców Wielkopolskich 17, 85-090 Bydgoszcz, Poland; katarzyna.kotarska@ibprs.pl

**Keywords:** mycotoxins, microorganisms, mold fungi, feed, health farm animals, biological detoxification

## Abstract

Secondary metabolic products of molds, called mycotoxins, negatively affect animal health and production. They constitute a significant problem in veterinary and medical sciences, and their presence has been confirmed in feed all over the world. Applying appropriate agricultural practices and ensuring proper storage conditions significantly reduces the contamination of agricultural products with mycotoxins. However, this does not guarantee that raw materials are completely free from contamination. Many detoxification methods are currently used, but their insufficient effectiveness and negative impact on the quality of the raw material subjected to them significantly limits their usefulness. The positive results of eliminating mycotoxins from many products have been proven by the specific properties of microorganisms (bacteria, yeast, and fungi) and the enzymes they produce. Biological detoxification methods seem to offer the most promising opportunities to solve the problem of the presence of mycotoxins in animal food. This work, based on literature data, presents the health risks to farm animals consuming mycotoxins with feed and discusses the biological methods of their purification.

## 1. Introduction

Mycotoxins (greek: “mycos”, latin: “toxicum”), are secondary metabolites produced by molds, exhibiting toxic effects on vertebrates [1] and insects [2]. The presence of fungal toxins has been confirmed in a number of plant foods [3,4,5] and animal foods (meat, eggs, and milk) [6,7,8,9]. They are widely present in products intended for animal feeding, like cereal grains, fruits, and vegetables [10,11,12]. It is assumed that about 360 species of mold fungi are able to produce toxic metabolites. The main producers of mycotoxins include the genera *Fusarium*, *Penicillium*, and *Aspergillus* [13]; this ability is genetically determined and related to basic metabolite pathways. Fungi strains may acquire or lose the ability to produce mycotoxins under specific environmental conditions, which indicates phenotypic conditioning [10,14,15,16]. Consumption of mycotoxins leads to a number of chronic and acute poisonings, negatively affecting livestock health and contributing to economic losses [17,18]. For this reason, measures are being taken to directly prevent the formation of mycotoxin-producing molds in agricultural crops in the field (crop rotation, tillage, sowing date, cultivation of varieties resistant to infection) [19,20,21] and at the storage stage (temperature, humidity, and cleanliness of storage facilities) [22,23,24,25]. Agricultural practices, agronomic treatments, and prevention at the plant growth stage are the basis for obtaining a contaminant-free yield [26]. These treatments do not guarantee complete purity of the raw materials for animal feed production. Therefore, other methods for detoxification of animal feed are being sought. Detoxification of animal feed involves neutralization or removal of the toxic substances contained in it. Physical and chemical treatments or feed additives incorporated into animal feed act as bio-transforming agents, converting the toxins into less toxic products without producing toxic residues or binding mycotoxins, so they are no longer bioavailable [27]. Physical methods of detoxification are used (segregation, washing, UV radiation, heat treatment, and adsorption on the carrier surface), and also with the use of chemical compounds (ammonia, ozone, cold plasma, hydrogen peroxide, acids, and bases) [28,29,30,31,32,33,34,35,36]. However, these measures are not sufficiently effective, often resulting in a loss of quality, nutritional value, and animal feed palatability. Hence, the latest research focuses on the use of microorganisms (bacteria, yeasts, and fungi) or their enzymes for the biological elimination of mycotoxin contamination [37,38].

Microorganisms can be obtained from various sources, such as soil, plants, animal digestive tracts, food products, and silage. These include lactic acid bacteria (*Lactobacillus*, *Bifidobacterium*, and *Streptococcus*), bacteria that naturally inhabit certain parts of the animal’s digestive tract, *Aspergillus* fungi, and *Saccharomyces cerevisiae* yeasts [16,39]. The neutralization of mycotoxins in feed takes place primarily through the adsorption of these compounds on the microorganism’s cell wall surface, mainly in the animal digestive tract, inhibiting the growth of mold, and changing the chemical structure of mycotoxin molecules to such an extent that they no longer have a harmful effect on animal organism [40,41].

Biological methods of mycotoxin decontamination seem to be an alternative to agronomic methods of limiting the formation of fungal toxins. Additionally, their use is effective and safe for the environment and animals fed with them, which has been confirmed by numerous studies presented in this review.

## 2. Effects of Mycotoxins on Animals Health and Their Characteristics

Feed containing mycotoxins consumed by animals leads to very negative health effects (Table 1). The contamination of fodders is most often associated with improper agrotechnical practices or failure to provide appropriate storage conditions for the raw materials. The World Food and Agriculture Organization (FAO) reports that as many as 25% of the world’s crops are contaminated with mycotoxins [42,43]. They can enter the animal’s body not only through food but also through the skin and mucous membranes, accumulating in internal organs (liver, kidneys, and muscles) [44,45,46]. These compounds differ in their chemical structures and effects on the organs of animals (Table 2) [14,18,47]. The growth stage of animals that are exposed to secondary fungal metabolites is most important. Young growing animals are more vulnerable to the harmful effects of mycotoxins than adult animals [48]. Negative effects of these compounds on dairy cattle [49,50], pigs [51], poultry [52], and even fish [53] have been reported.

## 3. Contamination of Animal Feed with Mycotoxins

The presence of mycotoxins in animal feed has been confirmed in research all over the world [62,63,64,65,66,67,68,69] (Table 3). These data indicate the problems of crop and animal feed contamination by mycotoxins, which require a solution. The vast majority of tested animal feed samples [63,64,65,66,68], compound feeds [67], and feed ingredients [62,69] contain at least one type of mycotoxin. The tests also showed that some of the analyzed samples exceeded the established limits for the amount of mycotoxins in the feed and raw materials used for their production. Every year, DSM-Firmenich company conducts a World Mycotoxin Survey concerning mycotoxin threats in livestock feed across the globe. The edition for January–December 2023 covers 95 countries and includes 113,558 analyses. In 61% of samples, multiple mycotoxins were detected, with particularly high risks observed in Europe and Eastern Europe, including deoxynivalenol, zearalenone, fumonisin, and toxin T-2 [70]. A comparison with the edition for January–December 2022, when 122,240 analyses were performed in 87 countries and 57% of the samples contained more than one mycotoxin, showed a significant increase in contamination across all major mycotoxins [71]. Therefore, it is important to regularly monitor levels of mycotoxins and prevent their occurrence.

The main mycotoxins that contaminate animal feed include deoxynivalenol (DON), nivalenol (NIV), T-2 and HT-2 toxins, aflatoxins, ochratoxin A (OTA), and zearalenone (ZEA) [72,73]. These mycotoxins are mainly produced by filamentous fungi belonging to the genera *Fusarium*, *Aspergillus*, *Penicillium*, *Alternaria*, and *Stachybotrys* [62,74]. The presence and amount of mycotoxins in animal feed can be determined using many laboratory techniques like liquid chromatography (LC), gas chromatography (GC), or immunoenzymatic testing (ELISA) [75]. These unfavorable research results clearly indicate the need for continuous monitoring of the production chain to ensure the appropriate quality of the produced fodder and the safety of humans consuming animal products. It should be noted that the presence of mycotoxins in animal feed, even in small quantities, poses a danger [76]. The synergistic effects of individual mycotoxins enhance their effects on the organs of animals. Various mycotoxins that interact with toxins of the same or different species in a living system can produce combined toxicity. Additive, antagonist, or synergistic toxicity can be induced by exposure to multiple mycotoxins [77,78].

## 4. Biological Detoxification of Animal Feeds

The most promising of the analyzed methods of mycotoxin elimination is the use of microorganisms (bacteria, yeasts, protozoa) [79] and their enzymes [80]. Their effectiveness has been confirmed at the stage of in vitro laboratory tests and directly in relation to the fodder that the animals were fed [40,81]. When analyzing methods for biological decontamination of mycotoxins, the COMMISSION REGULATION (EU) 2015/786 of 19 May 2015 defines acceptability criteria for detoxification processes applied to products intended for animal feed as provided for in Directive 2002/32/EC of the European Parliament and of the Council should be taken into account. This regulation contains a clear statement regarding the eligibility criteria for a (micro)biological detoxification process.

This review article includes both in vitro and in vivo studies. It should be mentioned that in vivo studies are relevant from a practical point of view, and in vitro studies are of scientific importance. However, the results of in vitro studies can be used for further in vivo investigations.

### 4.1. Reducing Deoxynivalenol by Microorganisms

Previously published research results have proven the effectiveness of deoxynivalenol (DON) degradation by several microorganisms from different sources (Table 4), such as soil, animal intestines, and plants [63,82]. Authors suggest that mycotoxin-transforming microorganisms may be present in catfish digestia (*Ameiurus nebulosus*) with frequent exposure to mycotoxins. Thus, environmental conditions may be significant determinants of their existence. Studies have shown that the toxicity of breakdown products such as DOM-1 is much lower compared with that of DON [83]. The 3-keto-DON showed a reduction of more than 90% in immunosuppressive toxicity compared with that of DON [84,85].

King et al. [86] determined that the cow’s rumen fluid converted all of the DON (up to 10 ppm) to the DOM-1 derivative within 24 h. The *Eubacterium* BBSH 797 strain is responsible for the possibility of transforming deoxynivalenol into less toxic compounds. This strain is currently the most extensively studied bacterial isolate and is capable of converting DON [63,87]. It was isolated from the cow rumen [88], and subsequent in vivo and in vitro studies have proven its effectiveness [89,90]. The presence of naturally occurring microorganisms in the digestive tract of ruminants and their ability to detoxify deoxynivalenol explain the lower sensitivity of these animals to the harmful effects of toxic metabolites. Monogastric animals like avians [43,52] are the most exposed to the harmful effects of deoxynivalenol due to the high proportion of cereals in their diet and the lack of a rumen with positive microbiota [58]. The toxicity of trichothecene mycotoxins results from their specific sesquiterpene structure, which contains one six-membered ring with an oxygen atom and an epoxide ring at positions 12 and 13. The deep oxidation reaction involving the intestinal flora and the pre-stomach, or in the presence of *Eubacterium* strains isolated from the rumen, reduces the toxicity of these mycotoxins [91]. These bacteria are added as feed additives under the trade name of the product *Biomin* ^®^ *BBSH 797*, which reduces the harmful effects of deoxynivalenol [91,92,93]. Sayyari et al. [94], however, indicate the insufficient effectiveness of the feed additive containing the bacterial strain *Coriobacteriaceum* DSM 11798 (the active ingredient of Biomin^®^ BBSH 797) in reducing deoxynivalenol contamination by acting on granulated feed. The authors point to the possibility of a difficult release process, effectiveness, or detoxification in the pig’s digestive tract. This suggests that the effectiveness of this preparation is limited to certain types of animal feed. Young et al. [95] conducted research on the biological detoxification of mycotoxins and determined that microbiota and pure cultures of microbial isolates obtained from chicken intestines showed the ability to degrade DON by deep oxidation and/or deacylation. Research is ongoing to use these results for the production of food for animals and fodders.

**Table 4:** Microorganisms used for deoxynivalenol degradation.

Microorganisms	The Source of the Microorganism	Degradation Product	Initial DON Concentration	DON Degradation Rate %	Experimental Medium/Buffer	Incubation Time	Source
Microbial culture C133 (*Ameiurus nebulosus*)	Fish intestines	DOM-1	50 μg/mL	100	FM medium	96 h	[83]
Devosia nanyangense DDB001	Soil (Nanyang, China)	No -determined	200 μg/mL	100	_	Three days	[96]
Microbial culture C20 (Hyphomicrobium genera et al.)	Wheat field (Jangsu, China)	3-keto-DON	70 μg/mL	100	MM medium	Five days	[85]
*Devosia insulae* A16	Soil (Nanyang, China)	3-keto-DON	20 mg/L	88	MM medium	48 h	[97]
*Pelagibacterium halotolerans ANSP101*	Seawater from the Bohai Sea	3-keto-DON	50 μg/mL	80	MMB2216 solid medium	12 h	[98]

### 4.2. Zearalenone Inactivation by Bacteria

#### 4.2.1. Zearalenone Binding by Bacteria

Zearalenone and its derivative α-zearalenol can be eliminated by *L. rhamnosus* bacterial strains through the adsorption of toxins to the peptidoglycan present on the surface of the bacterial cell wall [99,100]. According to Ćvek et al. [101], from the two tested strains *Lactobacillus rhamnosus GG (ATCC 5310)* and *L. plantarum A1*, *L. rhamnosus GG* was more effective in eliminating zearalenone (ZEA). Both strains were able to bind zearalenone at a significant concentration, and the degree of binding depended on the concentration of bacteria in the medium and incubation time.

#### 4.2.2. Zearalenone Biodegradation Effects

The research team of Lei et al. [102] tested the bacterial strain *Bacillus subtilis* ANSB01G isolated from the broiler’s digestive track and showed that it was able to reduce ZEA contamination by 88.65%. Under simulated swine gastrointestinal conditions, ANSB01G *B. subtilis* isolates led to the decomposition of 84.58%, 66.34%, and 83.04% of ZEA in naturally contaminated maize, dried grains, and mixed feed. The use of the *B. subtilis* strain was directly confirmed in a study conducted by Zhao et al. [103], which showed that the addition of strain ANSB01G *B*. *subtilis* to a diet of naturally contaminated ZEA fed to female pigs resulted in mitigation of the side effects caused by ZEA, keeping the sow organisms in a natural and healthy condition. A potential way to reduce the content of zearalenone may be the use of soil bacteria antagonistic to fungi of the *Fusarium* genus. Two strains of bacteria of the *Brevibacillus* genus used by Juś et al. [104] showed the ability to reduce the content of zearalenone by 12.7–80.9%.

### 4.3. Efficiency of Ochratoxin A Eliminating by Bacteria

The process of toxin reduction occurs by enzymatic pathways and adsorption on the bacteria cell wall surface. Selected lactic acid bacteria strains of the genera *Lactobacillus*, *Bifidobacterium,* and *Streptococcus*, as well as microorganisms isolated from the environment and the animal’s digestive tract (Table 5) [10,105], are capable of eliminating ochratoxin A. Each strain is characterized by a certain degree of ability to eliminate mycotoxins.

#### 4.3.1. Ochratoxin A Binding by Bacteria

Śliżewska and Piotrowska [106] indicate the potential of probiotic bacteria, including *Lactobacillus paracasei*, *Lactobacillus brevis*, and *Lactobacillus plantarum* in the reducing content of ochratoxin A from feed after 6 h of incubation. In addition, probiotic bacteria are resistant to gastric juice and bile, which limits the risk of bond breakdown and release of the toxin in the digestive tract of animals. Among the 30 strains of bacteria tested by Fuchs et al. [107] in terms of the binding efficiency of ochratoxin A, *L. acidophilus* VM20 turned out to be the most effective, binding 97% of the toxin. On the other hand, Luz et al. [108] demonstrated the binding efficiency over a wide pH range (3.5–6.5), pointing to the strains showing the highest efficiency, i.e., *L. rhamnosus* CECT 278T and *L. plantarum* CECT 749, which reduced OTA by 97% and 95%, respectively.

#### 4.3.2. Ochratoxin A Biodegradation

In addition to lactic acid bacteria, Mateo et al. [109] indicate the effectiveness of *Oenococcus oeni*, which degraded ochratoxin A contained in the culture medium in the presence of 2 µg OTA/L after 14-day incubation. The efficiency of OTA degradation has been demonstrated in both live and dead microorganism cells. Microbes naturally inhabiting the digestive system of ruminants can be used to eliminate OTA, making cattle less sensitive to mycotoxins than swine and poultry [10]. This microbiome consists mainly of protozoa capable of degrading OTA within 0.6–3.8 h. Studies have estimated that they are able to degrade 12 mg of ochratoxin/kg of animal feed, but under strictly defined, optimal conditions prevailing in the digestive tract [110].

**Table 5:** Degree of ochratoxin A degradation obtained with the use of selected microorganisms.

Microorganisms	The Source of the Microorganism	Degradation Product	Initial Mycotoxin Concentration	Degradation Rate %	Experimental Medium/Buffer	Incubation Time	Source
*Lactobacillus acidophilus* K1	Collection of cultures	Undefined	40 μg/mL	79	PBS medium	24 h	[111]
*Bacillus licheniformis* SI-1	Animal excrements	Undefined	Undefined	35	_	_	[112]
*Cupriavidus basilensis* Or16	Soil	OTα	20 μg/mL	100	LB medium	Five days	[113]
*Acinetobacter calcoaceticus* 396.1; *Acinetobacter* sp. Strain neg1	Soil taken from vineyards	OTα	10 μg/mL	82 91	MMS medium	6 days	[114]
*Stenotrophomonas* sp. CW117	Soil and food	Undefined	0.02 μg/mL	71	Corn and soybean feed	72 h	[115]
*Alcaligenes faecalis* ASAGF OD-1	Soil	OTα	1 μg/mL	92	LB medium	48 h	[116]
*Eubacterium biforme* MM11	Pig intestinal microflora	Undefined	1 ppm	100	Ground corn grains	24 h	[117]

### 4.4. Aflatoxins Binding or Degradation by Bacteria

The effectiveness of many species of bacteria in the elimination of aflatoxins contained in contaminated food products and at the stage of laboratory tests has been proven. Table 6 shows the degree of degradation or binding of aflatoxins by microorganisms, indicating the source of their origin. Literature shows that the mycotoxin degradation process leads to the formation of breakdown products with chemical properties different from the original molecules and total loss of mutagenicity [118]. Data on cytotoxicity tests have shown that biodegradable products are less toxic than pure AFB1 [119,120].

Lactic acid bacteria also play an important role as they can physically bind mycotoxins due to their appropriate cell wall structure [10,121,122]. Strains of the genus *Lactobacillus* sp., especially *L. plantarum*, show a special ability to remove aflatoxin B1 from cereal products and fodder [123,124]. Lahtinen et al. [125] estimated that peptidoglycan present in cell wall isolates derived from *L. rhamnosus GG cells* bound aflatoxin B1 (AFB1) from the experimental solution at 81%. The *Lactobacillus rhamnosus* LC-705 strain also showed high efficiency (~80% AFB1). The others from the group of tested strains, *Lactobacillus* spp. (*L. acidophilus* ATCC 4356, *L. gasseri* ATCC 33323, and *L. casei Sirota*) bound aflatoxin B1, with efficiencies ranging from 20% to 50% [10,99,100]. The minimum number of *Lactobacillus* bacteria that effectively bound aflatoxin B1, fumonisins B1 and B2, deoxynivalenol (DON), and zearalenone (ZEN) ranged from about 1 × 10^9^ cfu/cm^3^ to about 2 × 10^11^ cfu/cm^3^. The degree of toxin binding increased from 20% to 75% when the cell concentration increased from 10^8^ cfu/cm^3^ to 10^10^ cfu/cm^3^, respectively [99,100,126].

**Table 6:** The degree of aflatoxin degradation or binding was determined using the selected microorganisms.

Microorganism	Source of Origin the Microorganism	Degraded Mycotoxin	Initial Mycotoxin Concentration	Kind of Study	Degree of Degradation/Binding	Experimental Medium/Buffer	Incubation Time	Source
*Rhodococcus erythropolis*	Soil	AFB1	1.75 ppm	Degradation	66.8%	Liquid substrate	72 h	[118]
*Stenotrophomonas NMO-3*	Undefined	AFB1	100 μg/kg	Degradation	85.7%	_	_	[127]
*Streptococcus thermophilus* *Lactobacillus bulgaricus*	Yogurts	AFM1	50 μg/L	Binding	70% 87.6%	PBS liquid medium	14 h	[128]
*Pseudomonas putida MTCC1274*, *2445*	Undefined	AFB1	0.2 μg/mL	Degradation	100%	MSG medium	24 h	[119]
*Bacillus pumilus* *Enterobacter cloace*	Soil	AFB1	200 ppb	Degradation	88% 51%	Medium NB or MRS	10 days	[120]

These studies show that the ability to reduce the content of mycotoxins by bacteria of the genus *Lactobacillus* is a strain selective. Strains that reduce the content of one mycotoxin do not necessarily have to be effective in the degradation of another group of mycotoxins [129]. In addition to the aforementioned strains and bacterial abundance, mycotoxin concentration, temperature, pH, incubation time, cellular biomass preparation [99], and gastrointestinal conditions affect the efficiency of mycotoxin binding by bacteria. Data presented by Tajik and Sayadi [130] indicate that gastric juice and bacteria inhabiting the small intestine are involved in the reduction of AFB1 delivered with food. The process of aflatoxin adsorption is most effective in the presence of bacteria and gastrointestinal juice, whereas the environmental conditions in the saliva solution induce a low level of adsorption [131]. This feature is important because the most intensive absorption of mycotoxins occurs in the small intestine [132]. In addition, lactic acid bacteria inhibit the growth of *Monilia*, *Penicillium*, *Aspergillus*, and *Fusarium* fungi by producing fungicidal ingredients, such as organic acids, as well as competing for nutrients and through the synthesis of antagonistic compounds [133,134]. It has also been shown that heat-inactivated microorganisms have a higher affinity for mycotoxins and can bind them stably. In contrast, in living microbial cells, this process is reversible, which suggests a mechanism of toxin binding to cell wall elements and the interaction of electrostatic and hydrophobic forces [129]. Microbiological preparations containing lactic acid bacteria cultures can be used as feed additives, significantly reducing the negative impact of aflatoxins on livestock organisms and contributing to increased productivity and nutrient metabolism rates [135].

Aflatoxins can be metabolized by some Actinomycetales species, such as Nocardia corynebacterioides, Nocardia asteroids, Corynebacterium rubrum, Rhodococcus erythropolis, Mycobacterium fluoranthenivorans, and Mycobacterium smegmatis [63]. Sangare et al. [136] reported that Pseudomonas aeruginosa N17-1 can degrade aflatoxin AFB1 and AFB2 AFM1 by 82.8%, 46.8%, 31.9%, respectively, after incubation on NB medium at 37 °C for 72 h. Ali et al. [137] indicate the possibility of using the Pseudomonas fluorescens SZ1 strain isolated from poultry farms as an additive to feed mixtures, where the degradation efficiency of AFG1 was 100%, while that of AFB1, AFB2, and AFG2 was 99%. Some *Bacillus* sp. strains also show aflatoxin-reducing activity, such as the Bacillus subtilis UTBSP1 strain isolated by Farzaneh et al. [138] from pistachio nuts. Similar conclusions were reached by Gao et al. [139], who showed that the Bacillus subtilis strain from the fish gut has a strong ability to detoxify aflatoxins, and the percentage degradation of aflatoxins B1, M1, and G1 was 81.5%, 60%, and 80,7%, respectively. The bacterial isolate *Myxococcus fulvus* ANSMO68 derived from deer excrement was able to transform aflatoxin AFB1 to 80,7% in liquid medium. Liquid chromatography, mass spectrometry, and infrared analyses showed that AFB1 was transformed into a structurally different compound [140]. The isolate obtained by Shu et al. [141] also showed a stronger degradative activity of AFB1, amounting to 91.5%. This isolate was identified as the strain Bacillus velezensis DY3108, and the toxicity tests of the obtained products after degradation showed significantly (*p* < 0.05) lower cytotoxic effects than the parent AFB1. 

### 4.5. Reduction in Mycotoxins with Saccharomyces cerevisiae

Numerous experiments have confirmed the effectiveness of *Saccharomyces cerevisiae* in the reduction of bacterial and fungal toxins present in raw materials and fodders. It has been shown that the binding of mycotoxins takes place within 10 min of mixing with the product. Yeast-based preparations are also more economically viable. Yeast in the amount of 0.5 kg shows comparable effectiveness to 4 kg of mineral preparations based, for example, on aluminosilicates [129]. Similar to LAB, the mechanism of toxin removal involves adhesion to the cell surface [142]. The ability to remove mycotoxins is independent of the type of toxin, as demonstrated for zearalenone, patulin, T-2 toxin, and aflatoxin B1 [16,143,144]. The potential for toxin binding by yeast cells is due to the presence of β-D-glucan in their cell walls, particularly in its esterified form [16,145]. The hydroxyl, ketone, and lactose groups of toxins combine with β-D-glucan molecules by the generated hydrogen bonds and van der Waals interactions [16,146]. The ability to form complexes between yeast cells and toxins enables the use of yeast preparations as feed additives. This was confirmed in the studies by Yiannikouris et al. [147], who proved the effectiveness of aflatoxin binding by an adsorbent based on the yeast cell wall and limiting their bioavailability. Thus, mycotoxins are not absorbed but are removed from the animal’s body. The sorption properties of yeast range from 50 to 60% and depend on the type and concentration of fungal toxins, feed hydration, degree of fragmentation, and acidity in the animal’s digestive tract [129]. Adsorption of mycotoxins is most effective at acidic and close to neutral pH, i.e., those prevailing in some sections of the digestive tract. Alkaline conditions lead to changes in glucan structure, making adsorption impossible [16,146]. Changing to less favorable conditions when switching from acidic stomach conditions to more alkaline conditions may lead to the desorption of some mycotoxins like ochratoxin A [148]. Zhang et al. [149] showed that after 48 h of co-incubation, ZEA was completely removed by bound to the cell wall of *S. cerevisiae*. The authors suggest that the mechanisms involved in degradation may be associated with the production of intracellular and extracellular enzymes, the enhancement of yeast basal metabolism, and the production of functional proteins. Similar results were obtained by Keller et al. [150], where all *S. cerevisiae* yeast strains isolated from silages were able to remove 90% of zearalenone from the medium within two days. However, the elimination was mainly due to the biotransformation of ZEA to β-ZOL (53%) and α-ZOL (8%) rather than its adsorption on the yeast cell walls. It should be mentioned that β-zearalenone and α-zearalenone are toxic metabolites. Therefore, the degradation process in this case does not guarantee the production of feed that is safe for the health of farm animals [150]. Adsorbents based on the yeast cell wall consist of an insoluble carbohydrate fraction, thus retaining their properties along the entire length of the gastrointestinal tract [148,151]. Compared to the plant fraction, the binding of mycotoxins to the surface of yeast cells has also been demonstrated in aflatoxin elimination studies in food products. Rahaie et al. [152] conducted studies on pistachio nuts, and their results indicate aflatoxin binding on the surface of *S. cerevisiae* yeast cells at the level of 40% and 70% for the initial toxin concentration of 10 and 20 ppm, respectively. The authors indicate effectiveness for both live and dead yeast cells. Istiqomah et al. [153] determined that live *S. cerevisiae* B18 yeast cells showed a higher efficiency in binding aflatoxins (71.86%) than dead cells (69.52%) during 48 h of incubation. Chlebicz and Śliżewska [154] conducted research on the use of a probiotic, which included, among others, six yeast strains from the species *S. cerevisiae* to change the concentration of the most commonly detected mycotoxins in feed. A clear decrease in concentration was observed for all mycotoxins: 67–74% for the mixture of fumonisins B1 and B2, 65% for aflatoxin B1, 69% for T-2 toxin, and 52% for zearalenone. The lowest effectiveness of the probiotic was recorded for deoxynivalenol contamination, whose concentration decreased by only 22–43% after 24 h of incubation in PBS solution. Taran et al. [155] also demonstrated the efficacy of fumonisin elimination by *S. cerevisiae*. One of the strains showed higher adsorption values (at 1 × 10^9^ cfu/mL), adsorbing 39.4% of FB1 at pH 2 and 37.5% at pH 6.8, and all the tested strains showed an increasing level of adsorption at higher concentrations of AFB1 in corn grain. Oliveira et al. [156] suggest that the degree of mycotoxin binding is dependent on the *S. cerevisiae* strain, which would explain the differences in the reported results. The undoubted advantage of preparations based on yeast extracts is their easy biodegradability; they are completely safe for the environment, and no side effects are observed with their use, which are observed with mineral absorbents. Additionally, in animals fed fodder (e.g., broilers) enriched with *S. cerevisiae* yeast, in addition to the biodegradable effect of mycotoxins, improved growth, immunity, carcass characteristics, and no negative impact on their health were observed [157]. These studies indicate that the immobilization of yeast cells may increase the efficiency of their use in decontamination processes. Rahaie et al. [152] used immobilized *S. cerevisiae* yeast cells to eliminate aflatoxins. They confirmed that immobilized yeast cells showed higher resistance to unfavorable environmental conditions. This treatment can be commonly used in the future to eliminate the contamination of fodders with mold fungi metabolites.

### 4.6. Degrade Mycotoxins with Fungi

Many species of fungi show the ability to degrade mycotoxins. *Aspergillus* species are of particular importance here. Zhang et al. [158] isolated an *A. niger ND-1* strain that was capable of degrading aflatoxin B1. This demonstrated the effectiveness of removing the toxin from the NB medium by 58.2% after 24 h of using optimal culture conditions. A greater efficiency of AFB1 degradation was noted for the *A. niger* supernatant than for the cells and cell extracts. In addition, it has been found that aflatoxin degradation is a process that occurs in the extracellular environment, possibly through the secretion of digestive enzymes by fungal hyphae. Similar conclusions were reached by Sun et al. [159], who investigated the ability of *A. niger* FS10 strain to eliminate ZEA contamination in CSL (Corn Step Liquor). The strain showed an ability to remove the toxin by 89.56%, and the authors suggest that the strain’s filtrates can be safely used to remove toxic zearalenone from animal food and feed. Varga et al. [160] also report the ability to eliminate ochratoxin A from culture media by *A. niger* strains. Two fungal isolates of *Aspergillus tubingensis,* M036 and M074, isolated by Cho et al. [161], degraded OTA by over 95% after 14 days of incubation, and HPLC analysis showed that biodegradation of ochratoxin by A. *tubingensis* strains led to the production of α-ochratoxin, which is significantly less toxic than OTA. It is not the only *Aspergillus* sp. species that demonstrates the ability to eliminate mycotoxins. *Pleurotus ostreatus* (oyster mushroom), through the production of enzymes (lactase and manganese peroxidase), can degrade many environmentally hazardous compounds. This property was investigated by Das et al. [162] who evaluated the degradation efficiency of AFB1. The highest degradation was observed for *P. ostreatus* MTCC 142 (89.14%) and *P. ostreatus* GHBBF10 (91.76%), with an initial AFB1 concentration of 0.5 μg/1 mL. They have been proven effective in decontaminating naturally contaminated corn kernels by aflatoxin B1 to levels that are acceptable for livestock feed production. Moreover, the degradation products showed minimal mutagenicity compared to the parent form [163]. Branà et al. [164] indicate the possibility of using the *Pleurotus* erynii species to degrade AFB1 and purify the animal feed from mycotoxin. Tian et al. [165], who conducted research on the *Trichoderma* genus, indicated its ability to reduce zearalenone contamination by converting it to sulfated forms, as well as its antagonistic potential to control mycotoxin-producing pathogens. Brodehl et al. [166] prove in their studies that the *A. oryzae* strains and *Rhizopus* species were able to transform ZEA into various metabolites, including ZEN-14-sulfate, ZEN-O-14 and ZEN-O- 16-glucoside. Determination of the major metabolites indicated that more than 50% of the initial zearalenone concentration could be modified into less toxic forms. These results indicate the possibility of the effective use of certain species and strains of fungi to prevent the growth of toxin-causing molds on crops and the decontamination of contaminated batches of raw materials, resulting in clean, toxin-free feeds that can be fed to livestock.

### 4.7. Biodegradation of Mycotoxins with Enzymes

Enzymatic catalysis occupies a unique position among activities potentially suitable for mycotoxin elimination [37], and its use is more convenient due to the greater selectivity of the reaction [167]. Currently, feed additives that contain a composition that inactivates mycotoxins by enzymatic substances are used on a large scale. They are designed to break down fungal toxins that cannot be removed from an animal’s body by absorption into smaller fragments that no longer exhibit toxic effects [80].

#### 4.7.1. Mycotoxins Biodetoxification by Peroxidases

Noteworthy are peroxidases, whose effectiveness of mycotoxin elimination has been confirmed by studies. Manganese peroxidase (MnP), which is involved in cellulose breakdown, is able to degrade four major mycotoxins (AFB1, ZEA, DON, and FB1) in the presence of malonic acid. This enzyme can be used to detoxify raw materials and ready-made feed mixtures while monitoring the residual toxicity of degradation products [168]. Wang et al. [169] proved that MnP (5 nanocatals) is able to degrade AFB1 by 86% after 48 h of incubation. Aflatoxin B1 was first oxidized to AFB1-8,9-epoxide and then hydrolyzed to AFB1-8,9-dihydrodiol. The degradation products showed less toxicity as compared to the parent forms. It should be noted that endo-or exo-epoxides must be specified because aflatoxin B1 exo—8,9 epoxide is carcinogenic [170]. Garcia et al. [171] investigated the effectiveness of commercial peroxidases and peroxidases derived from soybean and rice bran in reducing the ZEA in a model solution. Commercial peroxidases reduced ZEA concentrations by 69.9%, while POD derived from soybean and rice bran reduced the concentration of ZEA by 47.4% and 30.6%, respectively, within 24 h. Feltrin et al. [172] used rice bran peroxidase to biodegrade deoxynivalenol. The DON reduction under optimal conditions was 20.3%.

#### 4.7.2. Enzymatic Biodetoxification of Fumonisins

Microbial degradation of fumonisins is initiated by the hydrolysis of ester bonds (e.g., via carboxylesterases) to its hydrolyzed HFB1 analogs, which are less toxic to swine [173]. Heinl et al. [174] characterized *Sphingomonas* spp. bacterium as a source of fumonisin detoxifying enzymes that can be used in feed decontamination, especially under limited oxygen conditions, e.g., in silage and in the animal’s digestive tract. Li et al. [175] obtained FumDSB carboxylesterase isolated from the *Sphingomonadales* bacterium, which catalyzes fumonisin B1 to its hydrolyzed form. However, there is no consensus regarding the toxicity of hydrolyzed fumonisin in living organisms. In addition to its high degradation ability, carboxylesterase has also been shown to be stable over a wide pH range (6.0–9.0) and moderately thermostability (30–40 Â °C). Fumonisin esteraze produced from a genetically modified strain of *Komagataella phaffi* (formerly *Komagataella pastoris*) degrades fumonisin B1 (FB1) and related fumonisins contaminants in the animal digestive tract. The active substance, the enzyme fumonisin esterase (EC 3.1.1.87), cleaves the diester bonds, releases tricarballylic acid, and is intended to reduce the toxicity of contaminated feed [176].

#### 4.7.3. Enzymatic Biodetoxification of Ochratoxins

Many enzymes may be involved in the microbial degradation of ochratoxin; however, few have been purified and characterized. Enzymes that degrade ochratoxins include hydrolase, protease A, carboxypeptidase Y [177]. One of the enzymes was isolated from *A. niger* by anion exchange chromatography. The metalloenzyme showed the ability to hydrolyze OTA at a reaction rate of Vmax = 0.44 microM/min. and Km = 0.05 mM, the reaction was carried out at 37 °C and pH 7.5 [178]. Filamentous fungi are an interesting and rich source of enzymes like hydrolases and proteases that are potentially capable of degrading ochratoxins. Among all the biochemical reactions that degrade ochratoxins, the enzymatic hydrolysis of the amide bond that links the coumarin ring and phenylalanine residue seems to be the most effective because it releases two harmless degradation products, ochratoxin α and L-β-phenylalanine [179].

#### 4.7.4. Enzymes Obtaining by Genetic Engineering Techniques

Innovative methods include the use of genetic engineering techniques to obtain enzymes with specific properties. Azam et al. [180] indicate the potential of using recombinant enzymes for the simultaneous degradation of several mycotoxins. Their recombinant bifunctional enzyme (ZHDCP) was created by combining two single genes, i.e., zearalenone hydrolase (ZHD) and *B. amyloliquefacines*-derived carboxypeptidase, and was able to completely degrade ZEA into a non-toxic product within 2 h (pH = 7; Temp. = 37 °C), with 100% degradation of OTA within 30 min (pH = 7; Temp. = 30 °C). This indicates the potential of using fusion and genetic engineering of single enzymes to obtain recombinants that can eliminate several different mycotoxins at the same time.

#### 4.7.5. Other Enzymatic Methods of Mycotoxins Biodetoxification

Enzymatic biodetoxification of mycotoxins is also possible with the participation of other enzymes. Most of the mycotoxin-detoxifying enzymes are obtained from the environment. Branà et al. [164,181,182], on the example of oyster mushroom (*Pleurotus* spp.) production, indicate the problem of wasting by-products of cultivation of mushrooms (*Pleurotus ostreatus*; *Pleurotus eryngii*) as a potential source of ligninolytic enzymes like laccase, Mn-peroxidase useful for AFB1 degradation. Reports by Tso et al. [183] indicate the effectiveness of enzyme degradation reagents (EDRs) in removing deoxynivalenol and zearalenone under in vitro simulated conditions of the pig and poultry digestive tract- synergy obtained by combining several compounds may have a positive effect. The enzymes that have shown the ability to degrade mycotoxins also include lactase isolated from *Trametes versicolor*, which eliminates aflatoxins B1 and M1 [184], the effectiveness of which has been confirmed in in vivo studies. Treatment of maize infected with *T. versicolor* culture filtrates containing lignolytic enzymes resulted in a significant reduction in aflatoxin B1 levels [185].

Despite the fact that enzymatic methods show effectiveness in the elimination of mycotoxins, their use in the detoxification of feed is limited, mainly due to the high cost of enzyme purification and acquisition of finished preparations.

## 5. Conclusions

This review paper presents a number of risks resulting from the failure to notice the problem of mycotoxins in animal feed. Therefore, the conclusions will also guide scientists and regulatory authorities or companies cultivating crops or producing feed regarding the need to take specific actions:

(1) There is a need to carry out agrotechnical treatments effectively and at the right time to prevent mold from developing in crops. This will reduce the need for other methods for detoxifying crops and animal feed.

(2) In the case of the occurrence of molds and secondary products of their metabolism (mycotoxins), microorganisms should be used, which, in addition to detoxifying mycotoxins, also increase the nutritional value of animal feed, such as *Saccharomyces cerevisiae*. Secondly, those that only enable the detoxification of feed.

(3) Since the limits set by the various regulatory authorities only apply to individual mycotoxins and do not take into account the joint occurrence of several mycotoxins (mixtures), there is a risk that the actual risk to the health and life of animals and—as a consequence of the consumption of animal products—of humans is underestimated.

(4) There is now an urgent need to create consortia of microorganisms that can effectively bind or biologically detoxify many mycotoxins and their degradation products simultaneously after addition to the feed.

(5) In order to consider a particular decontamination method or consortia of microorganisms as safe and effective, the toxicity of the resulting mycotoxin degradation products must be determined.

## Figures and Tables

**Table 1:** Classified negative health effects of consuming fodder contaminated with mycotoxins.

Species of Animals	Clinical Symptoms	Breeding Problems
Pigs (boars, sows)	Damage to the digestive tract and kidneys, vulva swelling and redness, rectal prolapse, interfere with cell function and signaling in many tissues, immunosuppression, liver dysfunction, such as hemorrhages, jaundice, in boars, suppresses testosterone levels [48,54,55,56].	Fertility problems, low libido, decreased productivity, fetal death, infections, reduced feed consumption, weight loss by reducing body fat, slow growth of piglets consuming mycotoxins with milk, vomiting, diarrhea, severe respiratory signs, with labored and openmouthed breathing, heart failure and fluid accumulation in the lungs, deaths [48,54,55,56].
Cattle (dairy cows, beef cattle)	Decreased globulin levels in serum, disturbances in protein synthesis, inhibition of DNA synthesis, inflammation and cirrhosis of the liver, inflammation of the kidneys, decreased blastogenesis of bovine lymphocytes, immunosuppression, and changes in liver cells [47,50,55].	Decreased milk production, reproductive dysfunction, loss of appetite, anorexia, intermittent diarrhea, other digestive upsets, unthriftiness, rough hair coat, and deaths [47,50,55].
Poultry (laying hens, ducks, broilers, turkeys)	Decreased intestinal integrity, gastric erosions, coccidiosis, immunodeficiency, organ changes including liver enlargement and dysfunction, focal hemorrhage, biliary tract hypertrophy, nodular lymphoid infiltrates, inactivation of enzymes responsible for starch breakdown, digestion of lipids and proteins, reduction of serum protein levels, anemia [43,52,55,57].	Decreased size, quality, and egg production increased susceptibility to infection, decreased productivity, poor pigmentation of skin, eggs, and yolk, growth retardation, abnormal feathering, and deaths [43,52,55,57].
Horses	Disturbances in the functioning of the nervous system (including leukoencephalomalacia), ataxia, paresis, apathy, impaired locomotor function, changes in the cerebral cortex and white matter necrosis in the brain, central nervous system dysfunction, increased heart rate [47,55].	Reduced feed intake or refusals, general lethargy, increased susceptibility to disease, altered heat cycles, and swollen mammary glands in mares deaths [47,55].
Sheeps	Changes and disorders of the liver and kidneys, metabolic disorders, cardiological disorders (atrial fibrillation), necrosis of the tongue and cheeks epithelial tissues, and disorders of DNA and protein synthesis [55,58].	Deaths, reduced fodder intake, reproductive dysfunction, and weight loss by reducing body fat [55,58].
Fishes	Anemia, impaired blood clotting, sensitivity to bruising, damage to the liver and other organs, and decreased immune responsiveness increase vulnerability to bacteria and viral or parasitic infections [59,60].	Decreased body weight, growth impairment, changes in swimming behavior, higher rates of disease, and mortality [59,60].

**Table 2:** The major species of farm animals that are endangered by molds producing mycotoxins that infect individual plant crops [10,14,18,37,57,61].

Mycotoxins	The Major Species of Molds Producing Mycotoxins	The Most Important Health Effects	Main Crops Threatened with Infestation	The Major Group Endangered Species of Farm Animals	Recommended Maximum Value of Mycotoxins in Feed Materials or Compound Feed Intended for Animal Feeding * (mg/kg)
Aflatoxins B1, B2, G1, G2	*Aspergillus flavus*, *A. parasiticus*, *A. nominus*	Carcinogenic, hepatotoxic, and teratogenic effects	Peanuts, nuts, corn, cotton seeds, wheat, barley, cocoa beans, dried fruit, spices	Poultry, pigs, fish,	Feed materials: 0.02 (AFB1) Complementary and complete feed: 0.005–0.01 (AFB1) **
Deoxynivalenol	*Fusarium graminearum*, *F. culmorum*, *F. poae*	Cytotoxic and immunosuppressive effects, digestive disorders, and reduced weight gain	Wheat, barley, corn, oats, rye, rice, grain products	Poultry, pigs, ruminants, fish	Cereals and cereal products: 8 Maize by-products: 12 Compound feed: 0.9–5
Fumonisins	*Fusarium moniliforme*, *F. proliferatum*, *F. verticillioides*, *F. subglutfinans*	Carcinogenic and hepatotoxic effects, pulmonary effects, encephalomalacia (brain necrosis) in horses	Corn, grapes	Pigs, horses	Maize and maize products (FB1 + FB2): 60 Compound feed (FB1 + FB2): 5–50
Ochratoxin A.	*Penicillum verrucosum*, *P. commune*, *P. nordicum*, *P. purpurescens*, *Aspergillus ochraceus*, *A. alutaceus*, *A. melleus*, *A. carbonarium*, *A. niger*	Carcinogenic, hepatotoxic, neurotoxic, nephrotoxin (kidney toxin) in pigs teratogenic, immunosuppressive effects, nephrotoxic effect	Grains, legumes, oilseeds, peanuts, cashews, dried fruit	Poultry, pigs	Cereals and cereal products: 0.25 Compound feed: 0.05–0.1
T-2 toxin; HT-2 toxin	*F. sporotrichioides*, *F. langsethiae* *F. poae*, *F. solani*	Digestive disorders, hematologic changes, negative influence on the immune system	Cereals	Poultry, pigs, ruminants, fish	Feed materials: 0.5 Complete feed: 0.25
Zearalenone	*Fusarium graminearum*, *F. culmorum*, *F. solani*, *F. cerealia*, *F. equiseti*	Estrogenic, potentially carcinogenic, and teratogenic activity, reproductive disorders	In all types of cereals, processed cereals, the highest levels in maize and wheat bran	Pigs, ruminants, lambs	Cereals and cereal products: 2 Maize by-products: 3 Compound feed: 0.1–0.5

Comments: * The recommended mycotoxin content in feed materials intended for animal feeding mg/kg (ppm), (2006/576/EC) (2013/165/EU), (574/2011/EC), (OJ L 140, 30.5.2002, p. 10). The recommended maximum content of mycotoxins in animal feed varies depending on the species and age of the farm animal. ** (2002/32/EC): maximum content in mg/kg (ppm) relative to a feed with a moisture content of 12%.

**Table 3:** Level of mycotoxin contamination in animal feed.

State/Region	Type of Animal Feed	Number of Tested Samples	Mycotoxin Detected	Pollution Degree %	Mycotoxin Concentration	Recommended Maximum Mycotoxin Content * mg/kg (ppm)	Source
Slovakia	Compound feeds for poultry	50	T-2 HT-2 ZEA DON	90 76 88 56	1–130 μg/kg 2–173 μg/kg 3–86 μg/kg 64–1230 μg/kg	0.25–0.5 (T-2 + HT-2) 0.1–0.5 5	[63]
Croatia	Grains and animal feeds	465	T-2 Diacetoxyscirpenol DON	16.8 27.6 41.2	0.05–3.4 mg/kg	0.25–0.5 (T-2 + HT-2) _ 0.9–5	[64]
Pakistan	Fodders ingredients, ready-made poultry feed	286 80	OTA	31 38	51 μg/kg 75 μg/kg	0.1	[65]
Poland	Fodders ingredients and ready-made feed mixtures	300	OTA	9	>0.03 μg/kg	0.05–0.1	[66]
Turkey (Sivas)	Compound feeds	89	OTA	71.91	5–>40 ppb	0.05–0.1	[67]
Uganda	Fodders from feed processing plants Fodders obtained from breeders	40 27	Aflatoxins	100	7.5–393.5 ppb 19–188.5 ppb	0.02 (AFB1) **	[68]
Croatia	Cereal grains (wheat, oats, corn, barley)	240	T-2 HT-2	33.8	82.7–96.5 μg/kg 83.6–94.1 μg/kg	0.25–0.5 (T-2 + HT-2)	[69]
Germany	Straw (wheat, barley, triticale, oats, rye)	192	ZEA DON T-2 HT-2	95.8	6.0–785 μg/kg 20–24,000 μg/kg 10–250 μg/kg 20–800 μg/kg	2–3 5 0.25–0.5 (T-2 + HT-2)	[62]

Comments: * The recommended mycotoxin content in feed materials intended for animal feeding mg/kg (ppm), (2006/576/EC), (2013/165/EU), (574/2011/EC), (OJ L 140, 30.5.2002, p. 10). The recommended maximum content of mycotoxins in animal feed varies depending on the species of farm animal and its age. ** (2002/32/EC): maximum content in mg/kg (ppm) relative to a feed with a moisture content of 12%.
